# Association between Chinese Dietary Guidelines Compliance Index for Pregnant Women and Risks of Pregnancy Complications in the Tongji Maternal and Child Health Cohort

**DOI:** 10.3390/nu13030829

**Published:** 2021-03-03

**Authors:** Ye Ding, Fangping Xu, Chunrong Zhong, Lishu Tong, Fang Li, Qian Li, Renjuan Chen, Xuezhen Zhou, Xiating Li, Wenli Cui, Yu Zhang, Li Huang, Shangzhi Xu, Chaoqun Liu, Jiangyue Wu, Xi Chen, Qin Gao, Nianhong Yang, Zhixu Wang

**Affiliations:** 1 Department of Maternal, Child and Adolescent Health, School of Public Health, Nanjing Medical University, Nanjing 211166, China; dingye@njmu.edu.cn (Y.D.); m18356581510_1@163.com (F.X.); tonglishu517@126.com (L.T.); LiFang7208@163.com (F.L.); 2 Department of Nutrition and Food Hygiene, Hubei Key Laboratory of Food Nutrition and Safety, MOE Key Laboratory of Environment and Health, School of Public Health, Tongji Medical College, Huazhong University of Science and Technology, Wuhan 430030, China; zchr0926@outlook.com (C.Z.); m201575210@hust.edu.cn (Q.L.); rjchen_es@126.com (R.C.); zxzluckly@icloud.com (X.Z.); tina31bd@hotmail.com (X.L.); savely-ok@163.com (W.C.); zhangyunew@126.com (Y.Z.); tjhuangli@126.com (L.H.); xushangzhi@shzu.edu.cn (S.X.); wendyliu919@163.com (C.L.); 13952401757@136.com (J.W.); chenxi_tj@hust.edu.cn (X.C.); gqnutr@163.com (Q.G.); zynh@mails.tjmu.edu.cn (N.Y.)

**Keywords:** maternal diet, dietary quality, Chinese dietary guidelines for pregnant women, gestational hypertension, gestational diabetes mellitus

## Abstract

Background: Compliance with dietary guidelines among pregnant women can positively influence not only their own health but also the health of their babies. Measuring the compliance requires professional skills in nutrition and dietary counseling. In China, few simple and effective techniques assess dietary quality among pregnant women, especially in rural areas. We aimed to establish a new simple and effective assessment technique, the “Chinese Dietary Guidelines Compliance Index for Pregnant Women (CDGCI-PW)” and assess the association between maternal dietary compliance and risks of pregnancy complications. Methods: The CDGCI-PW consists of 13 main components which were based on the 2016 edition of the Chinese dietary guidelines for pregnant women. Each component was assigned a different score range, and the overall score ranged from 0 to 100 points. The Tongji Maternal and Child Health Cohort study (from September 2013 to May 2016) was a prospective cohort study designed to examine maternal dietary and lifestyle effects on the health of pregnant women and their offspring. The maternal diet during the second trimester was compared with the corresponding recommended intake of the Chinese balanced dietary pagoda for pregnant women to verify their compliance with dietary guidelines. The association between maternal dietary quality and risks of pregnancy complications was estimated by regression analysis. Receiver operating characteristic (ROC) curves were constructed to identify the optimal cut-off values of CDGCI-PW for gestational hypertension and gestational diabetes mellitus (GDM). Results: Among the 2708 pregnant women, 1489 were eventually followed up. The mean CDGCI-PW score was 74.1 (standard deviation (SD) 7.5) in the second trimester. The majority of foods showed the following trend: the higher the CDGCI-PW score, the higher the proportion of pregnant women who reported food intake within the recommended range. Moreover, a higher maternal CDGCI-PW score was significantly associated with lower risks of gestational hypertension [odds ratio (OR) (95% confidence interval [(CI): 0.30 (0.20, 0.37)] and GDM [OR (95% CI): 0.38 (0.31, 0.48)]. The optimal CDGCI-PW cut-off value for gestational hypertension was ≥68.5 (sensitivity 82%; specificity: 61%; area under the ROC curve, AUC = 0.743), and the optimal CDGCI-PW cut-off score for GDM was ≥75.5 (sensitivity 43%; specificity: 81%; area under the ROC curve, AUC = 0.714). Conclusions: The CDGCI-PW is a simple and useful technique that assesses maternal diet quality during pregnancy, while adherence to the CDGCI-PW is associated with a lower risk of gestational hypertension and GDM.

## 1. Introduction 

Diet plays an important role in maternal health during pregnancy and can also influence birth outcomes and subsequent chronic disease risk in the offspring [1,2]. In China, the routine evaluation of diet quality is a complicated job that requires professional skills in nutrition clinics [3]. Specifically, information on the type and quantity of food that is consumed by individuals is obtained using 24-h dietary recalls, the food frequency questionnaire (FFQ), or other methods [4]. Thereafter, the intake of each category of food is calculated and compared with the recommended amount in Chinese balanced diet pagoda. Finally, energy and nutrient intake is calculated according to the Chinese food composition table and compared with Chinese dietary reference intakes. Dietary assessment as part of antenatal care for pregnant women is considered a challenge due to over-population and the limited access to dietitians [5]. In China, the maternal mortality ratio and the prevalence rates of gestational diabetes mellitus (GDM) and gestational hypertension vary greatly in different regions. The maternal mortality ratio in the western region is 118% higher than that in the eastern region [6], while the prevalence rates of GDM and gestational hypertension are higher in the northern region than that in the southern region [7,8]. This is closely related to the imbalance of social and economic development, which leads to the imbalance of medical allocation [9,10]. China’s first- and second-tier cities, which have access to more medical resources, can better ensure the health of pregnant women and infants [11]. However, the question remains: How can we provide the needed dietary guidance to pregnant women in other urban and rural areas? These mothers are not able to travel to big cities for antenatal care, and usually, there are no nutrition clinics in local maternal and child health centers or health service centers; furthermore, even if there are, studies indicate that practicing doctors lack necessary nutrition care competencies to provide dietary advice to their clients [12,13]. Therefore, it would be beneficial to establish a simple and effective technique to evaluate the dietary quality of pregnant women in China.

Since 2018, our research group has visited maternal and child health care centers in more than 20 rural areas in China to examine the establishment or development of pregnancy nutrition clinics. We identified the need for a simple and effective technique to evaluate pregnant women’s dietary quality. The existence of such a technique integrated into routine maternity care may assist in improving nutritional care, which may help pregnant women to make the right nutritional decisions. A dietary index is used to evaluate and quantify the diet of a population and analyze the relationship between dietary quality and certain health outcomes [14,15,16]. Recently, according to the dietary guidelines and dietary characteristics of populations in various countries, researchers have established several dietary indexes, such as the Healthy Diet Index (HEI) [14,15,17,18,19], the Dietary Quality Index (DQI) [16,20], and the Mediterranean Diet Score (MDS) [21,22]. In China, the Dietary Balance Index (DBI) [23,24] is most commonly used to evaluate the dietary quality of the general population. Slightly modified from the DBI, the Diet Balance Index for Pregnancy (DBI-P) was developed by Wang et al. for Chinese pregnant women of three trimesters [25]. The components of DBI-P are complex. The most prominent performance is that each component has a positive score or a negative score that reflects undernutrition and overnutrition problems, respectively; therefore, it is necessary to accurately estimate the intake of each food category and identify whether it is within the upper or lower limits of the recommended amount of that food. Therefore, it has not been effectively promoted and applied in hospitals. Currently, the application of DBI-P is limited to researchers. There are few studies on the use of DBI-P to evaluate the dietary quality of pregnant women; only in Chengdu in southwest China and Shaanxi and Lanzhou in northwest China [25,26,27]. Moreover, these studies only directly regard the score of DBI-P as the level of dietary quality and do not evaluate the representativeness of DBI-P on dietary quality and whether compliance with DBI-P affects the risk of pregnancy complications. 

Therefore, we aimed to establish a simple and easy dietary assessment, the Chinese Dietary Guidelines Compliance Index for Pregnant Women (CDGCI-PW), and investigate its representativeness on dietary quality and the association between maternal diet quality and the risk of pregnancy complications in the Tongji Maternal and Child Health Cohort. 

## 2. Materials and Methods

### 2.1. Development of CDGCI-PW 

#### 2.1.1. Components of CDGCI-PW

According to the 2016 edition of the Chinese dietary guidelines for pregnant women (CDG-PW) [1], several components of CDGCI-PW were developed to assess pregnant women’s dietary compliance throughout all trimesters. Authoritative experts were assigned to revise and score these components according to their significance, and eventually, 13 components were determined. However, due to limitations regarding the length of the article, only the CDGCI-PW in the second trimester was presented (Table 1), whereas components that correspond to other trimesters are shown in the Supporting Information (Tables S1 and S2). Components 1 and 2 measured whether the amount of variety in a person’s diet meets the recommendations of diversification specified in the CDG-PW. Components 3 to 12 measured the degree to which a person’s diet aligns with the recommendations of the CDG-PW for nine major food groups: staple food (cereals and their products, potatoes, and beans other than soybeans); whole grains and beans other than soybeans; green leafy and colored vegetables (red and yellow); milk and its products; soybean and its products; nuts; lean meat (livestock and poultry meat); animal blood and liver; and iodized table salt and iodine-rich seafood. Unhealthy diets, such as those including excessive intake of fat, sugar, and salt, may have short- and long-term health effects on pregnant women and their fetuses. Therefore, we included component 13 to measure a person’s healthy eating habits.

Food categories were based on the Chinese balanced dietary pagoda for pregnant women [1]. There are 11 food categories: staple food (cereals and their products, potatoes, and beans other than soybeans); vegetables; fruits; aquatic products (fish, shrimp, and shellfish); livestock and poultry meat; eggs; milk and its products; soybean and its products; nuts; vegetable cooking oil; and iodized salt (see Supporting information, Figure S1). The types of food (see Supporting information, Table S3) are based on the Chinese food composition tables [28]. Foods with the same name are considered the same type of food, as are foods made from the same ingredients, but prepared differently.

#### 2.1.2. Scoring Criteria for CDGCI-PW

Each component was assigned a different score range, and the overall index ranged from 0 to 100 points. Details of the scoring criteria are shown in Table 2. Food variety was the basic principle of a balanced diet. According to the dietary guidelines for Chinese residents [1], a balanced diet includes 11 categories of food per week and 12 kinds of food per day. Therefore, components 1 and 2 were of great importance; with scores ranging from 0 to 25 points, they accounted for a quarter of the total score. Components 3 to 12 were all scored based on whether the intake of food met the minimum recommendations of the Chinese balanced dietary pagoda for pregnant women [1]. The weights of components 3, 4, 6, 7, 8, and 11 were the same, with scores ranging from 0 to 4 points. According to CDG-PW, pregnant women should consume iron-rich foods, iodized table salt, ensure the intake of necessary carbohydrates, and appropriately increase the intake of lean livestock meat, poultry, aquatic products (fish, shrimp, and shellfish), eggs, milk and its products. Therefore, the weights of components 5, 9, 10, and 12 were increased, with a maximum score of five points. Component 13 measured poor eating habits, involving pickled food, fried food, cream cake, chocolate, and other high-salt, high-oil, and high-sugar food. The consumption of these foods more than three times per week on average indicated poor eating habits and scored as 0 points; otherwise, the score was 6 points.

### 2.2. Study Population

All study participants were from the Tongji Maternal and Child Health Cohort study, a prospective cohort study designed to examine maternal dietary and lifestyle effects on the health of pregnant women and their offspring [29]. From September 2013 to May 2016, pregnant women in the first trimester (4–12 weeks) were recruited from maternity clinics in three public hospitals in Wuhan, the largest city in central China. General information was collected via questionnaire-based interviews at enrollment, and information on lifestyle and dietary intake was obtained at the follow-up visits made during middle pregnancy. This study was approved by the Ethics Committee of Tongji Medical College of Huazhong University of Science and Technology (NO. 201302) and registered at clinicaltrials.gov as NCT03099837. All participants provided written informed consent. Healthy women with singleton pregnancies, aged 18–45, with information on dietary intake, and no pregnancy complications were eligible for inclusion in the present study. Pregnant women who could not report their dietary intake because of limited cognitive capacity and pregnant women who underwent in vitro fertilization, chemotherapy, or were administered psychotropic drugs were excluded.

### 2.3. Processes of Data Acquisition

During recruitment in the first trimester, pregnant women completed a basic information questionnaire, which included age, pre-pregnancy weight, occupation, education level, household monthly income, parity, and telephone number. Height and weight were measured by a trained investigator at the same time. Pre-pregnancy body mass index (BMI) was calculated as self-reported pre-pregnancy weight divided by the squared height (kg/m^2^).

At 16–20 weeks of gestation, dietary information was obtained through FFQ. This FFQ was a semi-quantitative questionnaire, which consists of three parts, including the food list, frequency of eating a certain food, and amount of food consumed each time. There were 61 items on the food list, divided into 13 categories: staple food (cereals and their products, potatoes, and beans other than soybeans); vegetables; fruits; aquatic products (fish, shrimp, and shellfish); livestock meat and poultry; eggs; milk and its products; soybean and its products; nuts; cooking oil; processed food; flavorings (sugar, salt, monosodium glutamate, soy sauce, etc.); and beverages. This FFQ was used to collect the dietary intake of pregnant women over the previous 28 days and has been tested for reliability and validity [30]. The information was collected in person by trained graduate students majoring in nutrition. To improve the accuracy of food-weight estimation, we used a photographic atlas of food portions developed by our research team for use in dietary intake recall [31]. A standard 75-g 2-h oral glucose tolerance test was conducted at 24–28 weeks of gestation. GDM was diagnosed according to the criteria of the International Association of Diabetes and Pregnancy Study Groups in cases when any point of plasma glucose values exceeded the cut-off values: ≥5.1 mmol/L at fasting, ≥10.0 mmol/L at 1 h, and ≥ 8.5 mmol/L at 2 h [7]. Standard body weight and blood pressure measurement were performed at each visit. As recommended by the American College of Obstetricians and Gynecologists, chronic hypertension was defined if blood pressure (BP) was elevated at or before 20 weeks of gestation. Gestational hypertension was diagnosed if BP ≥ 140/90 mmHg after 20 weeks of gestation [32].

### 2.4. Statistical Analysis

Maternal dietary data obtained by FFQs at 16–20 weeks of gestation were used to calculate the CDGCI-PW scores according to the method described above. To verify the accuracy, original data were entered twice into the EpiData software program by two trained graduate students majoring in nutrition. The data were then exported to an Excel spreadsheet to calculate daily food intake and CDGCI-PW scores. Statistical analysis of all data was performed using SPSS V.26. Normally distributed continuous variables were expressed as the mean and standard deviation (SD) and non-normally distributed continuous variable data were expressed as median and interquartile range. Categorical variables were expressed as frequency (*n*) and percentage (%).

The intake of foods in nine major food groups (staple food; vegetables; fruits; aquatic products; livestock meat and poultry; eggs; milk and its products; soybean and its products; nuts) was compared with the corresponding recommended intake in the Chinese balanced dietary pagoda for pregnant women [1] to verify the representativeness of CDGCI-PW on dietary quality. According to the quartile of CDGCI-PW scores, the number of pregnant women within and out of the intake recommendation ranges and maternal characteristics were calculated. *p*-values were assessed using analysis of variance for normally distributed continuous variables and the chi-square (χ^2^) test for categorical variables. Two-sided *p*-values < 0.05 were considered as significant.

To study the relationship between CDGCI-PW score and pregnancy complications, we re-assigned CDGCI-PW scores for every 10-point increment. The association between the CDGCI-PW score and pregnancy complications were examined by logistic regressions. The models were adjusted for maternal age, pre-pregnancy BMI, weight gain in the second trimester, educational level, and household income.

The receiver operating characteristic (ROC) curve was carried out to determine the optimal cut-off values of CDGCI-PW score for gestational hypertension and GDM with the maximum Youden index (sensitivity + 1-specificity). The areas under the ROC curve (AUC) revealed the reliability as predictive markers for gestational hypertension and GDM and the AUC values indicated the predictive power of the model, as follows: >0.9, very good; >0.8, good; and >0.7, useful [33].

## 3. Results

### 3.1. Participant Characteristics

In this study, 1489 pregnant women provided complete dietary data. The mean ± SD of the CDGCI-PW score was 74.1 ± 7.5 (range: 46–94). The average maternal age was 28.4 years, and more than 85% of pregnant women were giving birth for the first time. As shown in Table 3, women with higher CDGCI-PW scores tended to be taller and had higher levels of education and household incomes; however, no trend was observed for age, pre-pregnancy BMI, weight gain in the second trimester, weight gain throughout pregnancy, or parity. 

### 3.2. Food Intakes according to CDGCI-PW Scores

The diversity of food intake is the basic principle of a balanced diet. Pregnant women with higher CDGCI-PW scores tended to consume more kinds of food every day. According to the quartile of CDGCI-PW scores ranged from low to high, women consumed 11.1 ± 3.9, 11.7 ± 4.4, 14.1 ± 4.7, and 15.3 ± 4.5 kinds of food, respectively. As shown in Table 4, almost all foods showed the following trend: the higher the CDGCI-PW score, the higher the proportion of pregnant women reporting food intake within the recommended range. The proportions of pregnant women in the first quartile of CDGCI-PW scores with intakes of staple food, vegetables, fruits, aquatic products, livestock meat and poultry, eggs, milk and its products, soybean and its products, and nuts lower than the recommended intakes were 78.6%, 54.4%, 18.8%, 84.0%, 56.9%, 71.1%, 86.4%, 80.0%, and 61.9%, respectively. However, the proportions of pregnant women in the fourth quartile of CDGCI-PW scores with intakes of foods mentioned above decreased to 58.5%, 25.6%, 8.4%, 16.4%, 18.4%, 49.0%, 38.3%, 59.9%, and 16.4%, respectively. 

### 3.3. Associations between CDGCI-PW Scores and Risks of Pregnancy Complications

Among the pregnant women in this study, 71 (4.8%) had gestational hypertension, and 163 (10.9%) had GDM. Statistical analysis of the data showed that higher CDGCI-PW scores in the second trimester were associated with lower risks of gestational hypertension [OR (95% CI):0.30 (0.20, 0.37); *p* < 0.01] and GDM [OR (95% CI): 0.38 (0.31, 0.48); *p* < 0.01] (Table 5).

**Figure 1:**
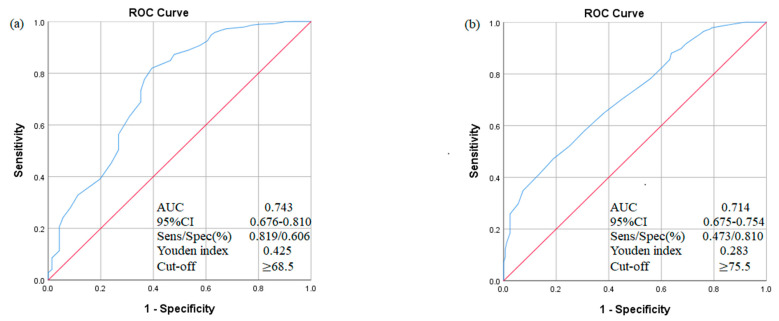
ROC curve for CDGCI-PW score in predicting gestational hypertension (**a**) and GDM (**b**). ROC curve, receiver operating characteristic curve; AUC, area under the ROC curve; CI, confidence interval; Sens, sensitivity; Spec, specificity.

The performances of the CDGCI-PW score were useful in predicting gestational hypertension and GDM among pregnant women. The optimal CDGCI-PW cut-off score for gestational hypertension was ≥ 68.5 (sensitivity 82%; specificity: 61%; area under the ROC curve, AUC = 0.743), and the optimal CDGCI-PW cut-off score for GDM was ≥ 75.5 (sensitivity 47%; specificity: 81%; area under the ROC curve, AUC = 0.714) (Figure 1). 

### 3.4. Sensitivity Analyses

**Figure 2:**
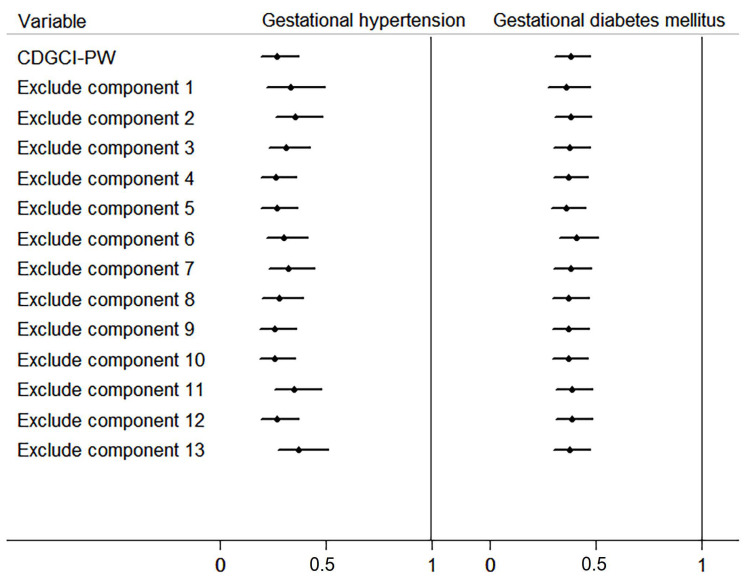
Association between maternal CDGCI–PW scores alternately excluding individual components and risks of pregnancy complications (per 10-point increment). Black dots for gestational hypertension and gestational diabetes mellitus denote logistic regression coefficients and horizontal lines denote 95% CIs. CDGCI–PW: Chinese dietary guidelines compliance index for pregnant women. Component 1, food categories per week on average. Component 2, food kinds per day on average. Components 3 through 12, the degree to which a person’s diet accords with the recommendations of the CDG-PW for nine major food groups: staple food (cereals and their products, potatoes, and beans other than soybeans), whole grains and beans other than soybeans, green leafy and colored vegetables (red and yellow), milk and its products, soybean and its products, nuts, lean meat (livestock and poultry meat), animal blood and liver, iodized table salt, and iodine-rich seafood. Component 13, eating habits.

According to the association between CDGCI-PW scores and the risk of pregnancy complications, the CDGCI-PW scores were redistributed for every 10-point increment to investigate the relationship between the CDGCI-PW scores and gestational hypertension, GDM. In this model, we adjusted for maternal age, pre-pregnancy BMI, weight gain in the second trimester, educational level, household income, and excluded 13 components of the CDGCI-PW in turn. We found that alternately excluding one component of the CDGCI-PW score did not substantially change the association between CDGCI-PW scores and the risk of pregnancy complications (Figure 2). 

## 4. Discussion 

In this study, we developed a simple and easy CDGCI-PW scoring system based on the latest version of the CDG-PW. The dietary quality of pregnant women in the second trimester from Tongji Maternal and Child Health Cohort was scored using this scoring system. We found that the higher the CDGCI-PW score, the better the dietary quality. Furthermore, a higher maternal diet quality was significantly associated with a lower risk of gestational hypertension and GDM. 

Each kind of food has its own nutritional characteristics, such as staple foods, which mainly provides carbohydrates, vegetables, which can provide rich dietary fiber, and meat, which can provide high-quality protein. The intake of a variety of foods ensures a wider range of nutrients for the pregnant woman’s body and the growing fetus. Therefore, the diversity of food intake is the basic principle of a balanced diet. The CDGCI-PW scoring system examined whether the categories and types of food in the diet were diversified or not. However, the previously developed DBI-P in China did not take this into account [24,34,35]. Recall of food categories and types was easier and simpler than estimating food weight. Our scoring system also evaluated food intake but differed from a previous method that set different scores according to the amount of food intake [14,15]. It is difficult for individuals to estimate food intake without a reference system and sufficient time. The CDGCI-PW mainly considers food that has an important impact on the health of pregnant women, and whether its intake frequency and amount are in line with the recommendations contained in the Chinese balanced dietary pagoda for pregnant women. The frequency of food intake is an easy question to answer. There were only nine kinds of food included in our method. Hence, we questioned pregnant women whether the food they consumed met the minimum recommended value, instead of asking them to estimate the quantities of each kind of food they consumed. Each minimum recommended quantity can be displayed in the form of food models or food atlas [31] as references. Due to the diversity and complexity of traditional Chinese cooking methods and the influence of Western fast food and pastries, the actual intake of oil, salt, and sugar of Chinese people cannot be accurately estimated in general. Previous methods for pregnant women rarely considered this issue [18,23]. In the CDGCI-PW system, this information was obtained by simply asking about the women’s eating habits. Moreover, our scoring system did not set an upper limit for food intake. The main reason for this was that our dietary assessment was designed as part of a health management program during pregnancy. In the Chinese maternal and child health care system, there are routine prenatal examinations and weight checks. If a healthy pregnant woman’s food intake exceeds the upper limit, it will be reflected in her weight gain. This scoring method serves as a dietary screening tool during pregnancy. Once dietary problems are identified, health care providers can direct pregnant women to a specific hospital, where doctors can further collect detailed dietary information and provide further assessment and guidance. Our research group has developed a WeChat app for CDGCI-PW, which includes weight management modules and the corresponding dietary suggestions. In the past two years, we have trained more than 3000 doctors or nurses who are engaged in the maternity nutrition clinics of Chinese primary hospitals and support that the CDGCI-PW is a very simple and convenient technique. The time required for doctors or nurses to complete the assessment of one pregnant woman is about 5–10 min, which is much less than the time required for routine 24-h dietary recall of at least half an hour. Through the above description, it is apparent that the method is scientific, reasonable, and simple enough for doctors in remote areas of China to evaluate the diet of pregnant women. Moreover, its simplicity and ease of use do not reduce its effectiveness for the evaluation of dietary quality. We have previously used the CDGCI-PW to evaluate the dietary quality of 160 pregnant women in the first, second, and third trimester of pregnancy (data not shown). The results are similar to those obtained from 1489 women in the second trimester of this study. The higher the CDGCI-PW score, the higher the proportion of pregnant women who consume foods beyond the lower limit of the recommended range, which indicates that the score is representative of the overall dietary quality. Therefore, the CDGCI-PW scoring system is suitable to evaluate the dietary quality of Chinese pregnant women. 

Using the CDGCI-PW, we investigated the association between maternal diet quality during the second trimester and the risk of pregnancy complications in the Tongji Maternal and Child Health Cohort. Gestational hypertension and GDM are common complications during pregnancy that seriously threaten the health of mothers and their infants. It is estimated that the prevalence of gestational hypertension and GDM in China is approximately 7.6% and 13%, respectively [7,8]. Several studies showed that maternal dietary quality was associated with gestational hypertension [36,37] and GDM [38,39]. Hence, improving the quality of mothers’ diets plays a positive role in reducing the occurrence of these complications [40,41,42,43,44]. Our results are similar to those of these studies; the higher the pregnant woman’s CDGCI-PW score, the higher her diet quality and the lower her risks of gestational hypertension and GDM. Moreover, the CDGCI-PW reflects the core recommendations of the CDG-PW in the form of a whole. The Forest plot of our sensitivity analysis revealed that excluding any component of the CDGCI-PW scoring system did not change the relationships between scores and outcomes. This indicated that the 13 components were reasonably set up and that no component played a leading role. However, the scoring systems designed in previous studies did not examine the role of individual components in the total score [45,46,47]. Therefore, the representativeness of the scoring systems used in these studies for maternal dietary quality required further confirmation.

Our research had some limitations. During the design, implementation, and data collection of this cohort study, we implemented quality control measures to minimize any deviation. During the use of FFQ to collect dietary information, we used the quantitative food atlas [31] and a face-to-face dietary survey to help pregnant women recall the types of food they consumed and improve the accuracy of food weight estimation. However, the FFQ was very complex, and focused on a special group of pregnant women. Therefore, whether it is the acquisition of dietary information involved in this cohort or the later use of CDGCI-PW to evaluate dietary quality, there may be some social desirability bias or other systematic biases that may influence the data reporting and association between diet and risks of pregnancy complications. 

## 5. Conclusions

In conclusion, we observed that CDGCI-PW scores reflected pregnant women’s adherence to the CDG-PW, and that dietary adherence was associated with lower risks of gestational hypertension and GDM in the Tongji Maternal and Child Health Cohort. Based on our findings, we suggest that the CDGCI-PW can be used in maternity nutrition clinics of Chinese primary hospitals in the future. We also hope that our research will help in guiding prenatal care changes in Chinese maternal and child health care systems to overcome malnutrition in pregnant women. The CDGCI-PW is based on the Chinese dietary guidelines for pregnant women, so its impact is limited to China, but other countries should also use this similar method to think about how to establish a suitable, more convenient, and efficient dietary assessment method.

We believe that the CDGCI-PW can be used in combination with pregnancy weight management and routine pregnancy health care, to effectively, simply, and quickly carry out the work of pregnancy nutrition clinics in the future.

## Figures and Tables

**Table 1:** Key recommendations of dietary guidelines for pregnant women and components of CDGCI-PW.

Key Recommendation	Components of CDGCI-PW
(1) Eat a variety of foods, mainly cereals and their products (a balanced diet includes 11 categories of foods per week and 12 kinds of foods per day. Whole grains and beans other than soybeans should form no less than one-third of the total intake of staple food).	1. How many categories of food do you eat per week on average?
	2. How many kinds of foods do you eat per day on average?
	3. Can whole grains and beans other than soybeans account for more than one-third of your staple food intake?
(2) Eat a balanced diet with no less than 130 g of carbohydrates per day.	4. Does your daily intake of staple food reach 200 g on average?
(3) Ensure adequate intake of vegetables, milk and its products, soybeans and its products, and nuts. Among them, the intake of green leafy and colored vegetables (red and yellow) should reach 200 g per day.	5. Do you eat more than 200 g of green leafy and colored vegetables (red and yellow) per day on average (raw weight)?
	6. How often do you drink milk and its products per week on average? Dairy intake is considered to be significant when servings are equivalent to 250 mL of fresh liquid milk each time.
	7. How often do you eat soybeans and soybean products per week on average? The intake of soybeans products is considered to be significant when servings are the equivalent of up to 15 g of dry soybeans each time.
	8. How often do you eat nuts per week on average? Nuts intake is considered to be significant when servings are the equivalent of up to 10 g of dry nuts each time.
(4) Eat appropriate amounts of lean meat (livestock and poultry meat), aquatic products (fish, shrimp, and shellfish), and eggs. Eat iron-rich foods such as animal blood or liver once or twice a week.	9. Do you eat 125 g of lean meat (livestock and poultry meat), aquatic products (fish, shrimp, and shellfish), or eggs per day on average?
	10. How often do you eat animal blood and liver per week on average? The intake of animal blood and liver is considered to be significant when it is reach to 20 to 50 g each time.
(5) Eat iodized table salt and iodine-rich seafood.	11. Do you eat iodized table salt every day?
	12. How often do you eat iodine-rich seafood per week on average? This includes kelp, nori, undaria pinnatifida, shellfish, sea fish, etc.
(6) Develop healthy eating habits	13. Do you often eat foods high in oil, salt, and sugar?

CDGCI-PW: Chinese dietary guidelines compliance index for pregnant women.

**Table 2:** Scoring criterion of CDGCI-PW.

Components	Scoring Criterion	Range of Score
1. How many categories of food do you eat per week on average?	A. Less than or equal to 6 categories	0 points
	B. Range from 7 to 10 categories	5 to 20 points (5 points for each additional category)
	C. More than or equal to 11 categories	25 points
2. How many types of foods do you eat per day on average?	A. Less than or equal to 4 kinds	0 points
	B. Range from 5 to 11 kinds	1 to 7 points (1 point for each additional kind)
	C. Range from 11 to 20 kinds	7 to 25 points (2 points for each additional kind)
	D. More than or equal to 20 kinds	25 points
3. Can whole grains and beans other than soybeans account for more than one-third of your staple food intake?	A. No	0 points
	B. Yes	4 points
4. Does your daily intake of staple food reach 200 g on average?	A. No	0 points
	B. Yes	4 points
5. Do you eat more than 200 g of green leafy and colored vegetables (red and yellow) per day on average? (raw weight)	A. No	0 points
	B. Yes	5 points
6. How often do you drink milk and its products per week on average? Dairy intake is considered to be significant when servings are equivalent to 250 mL of fresh liquid milk each time.	A. Less than or equal to once a week	0 points
	B. Range from 2 to 4 times per week	1 to 3 points (1 point for each additional time per week)
	C. More than or equal to 5 times per week	4 points
7. How often do you eat soybeans and soybean products per week on average? The intake of soybeans products is considered to be significant when servings are the equivalent of up to 15 g of dry soybeans each time.	A. Less than or equal to once a week	0 points
	B. Range from 2 to 4 times per week	1 to 3 points (1 point for each additional time per week)
	C. More than or equal to 5 times per week	4 points
8. How often do you eat nuts per week on average? Nuts intake is considered to be significant when servings are the equivalent of up to 10 g of dry nuts each time.	A. Less than or equal to once a week	0 points
	B. Range from 2 to 4 times per week	1 to 3 points (1 point for each additional time per week)
	C. More than or equal to 5 times per week	4 points
9. Do you eat 125 g of lean meat (livestock and poultry meat), aquatic products (fish, shrimp, and shellfish), or eggs per day on average?	A. No	0 points
	B. Yes	5 points
10. How often do you eat animal blood and liver per week on average? The intake of animal blood and liver is considered to be significant when it reaches 20 to 50 g each serving.	A. Never	0 points
	B. 1 time per week	3 points
	C. More than or equal to 2 times per week	5 points
11. Do you eat iodized table salt every day?	A. No	0 points
	B. Yes	4 points
12. How often do you eat iodine-rich seafood per week on average? This includes kelp, nori, undaria pinnatifida, shellfish, sea fish, etc.	A. Never	0 points
	B. Range from 1 to 4 times per week	1 to 4 points (1 point for each additional time per week)
	C. More than or equal to 5 times per week	5 points
13. Do you often eat foods high in oil, salt and sugar?	A. Yes	0 points
	B. No	6 points

CDGCI-PW, Chinese dietary guidelines compliance index for pregnant women.

**Table 3:** Characteristics of 1489 participants according to quartile of the CDGCI-PW Scores ^1^.

Characteristics	Value				*p*-Value
	First Quartile (*n* = 425)	Second Quartile (*n* = 331)	Third Quartile (*n* = 386)	Fourth Quartile (*n* = 347)	
CDGCI-PW score ^2^	66.0 (63.0, 69.0)	72.0 (71.0, 74.0)	77.0 (76.0, 78.0)	83.0 (81.0, 86.0)	
Age, y	28.3 ± 3.5	28.2 ± 3.3	28.4 ± 3.6	28.6 ± 3.5	0.41
Pre-pregnancy BMI, kg/m^2^	21.1 ± 2.9	21.1 ± 2.8	20.9 ± 2.8	20.7 ± 2.5	0.12
Height, cm	159.6 ± 5.1	159.8 ± 4.5	160.7 ± 4.7	160.9 ± 5.4	<0.01
Weight gain in the second trimester, kg	7.9 ± 3.6	8.6 ± 3.5	8.4 ± 3.5	8.4 ± 3.5	0.07
Weight gain throughout pregnancy, kg	15.5 ± 4.4	16.6 ± 4.7	16.1 ± 4.5	16.2 ± 4.7	0.17
Educational level, %					<0.01
≤9	4.2	2.4	2.3	1.4	
10–12	14.1	10.6	8.7	7.8	
13–15	31.1	58.5	27.0	23.1	
≥16	50.6	28.5	62.0	67.7	
Household income, % (Yuan/month, 1¥ = 0.15$)					<0.01
≤1000	1.2	0	0.8	0.2	
1001–2999	10.4	6.3	5.2	5.2	
3000–4999	39.5	38.7	30.8	26.8	
5000–9999	36.2	41.7	47.4	45.0	
≥10,000	12.7	13.3	15.8	22.8	
Primiparous, %	16.5	13.3	15.5	13.8	0.59

^1^ Values are presented as means ± SDs unless otherwise specified. *p*-values were assessed by using analysis of variance of the quartile for normally distributed variables or using χ^2^ test for categorical variables. CDGCI-PW, Chinese dietary guidelines compliance index for pregnant women. ^2^ Median (IQR).

**Table 4:** The food categories intakes of 1489 participants according to quartile of CDGCI-PW scores ^1^.

Food Categories	First Quartile (*n* = 425)			Second Quartile (*n* = 331)			Third Quartile (*n* = 386)			Fourth Quartile (*n* = 347)			Rec ^5^ (g/d)	*p*- Trend
	Below Rec ^2^	Within Rec ^3^	Above Rec^4^	Below Rec ^2^	Within Rec ^3^	Above Rec ^4^	Below Rec ^2^	Within Rec ^3^	Above Rec ^4^	Below Rec ^2^	Within Rec ^3^	Above Rec ^4^		
Cereals and their products, potatoes and beans other than soybeans	334 (78.6)	50 (11.8)	41 (9.6)	236 (71.3)	58 (17.5)	37 (11.2)	253 (65.5)	62 (16.1)	71 (18.4)	203 (58.5)	72 (20.7)	72 (20.7)	275–325	<0.01
Vegetables	231 (54.4)	144 (33.9)	50 (11.8)	155 (46.8)	107 (32.3)	69 (20.8)	135 (35.0)	153 (39.6)	98 (25.4)	89 (25.6)	134 (38.6)	124 (35.7)	300–500	<0.01
Fruits	80 (18.8)	183 (43.1)	162 (38.1)	54 (16.4)	139 (42.0)	136 (41.4)	41 (10.6)	161 (41.7)	184 (47.7)	29 (8.4)	135 (38.9)	183 (52.7)	200–400	<0.01
Aquatic products	357 (84.0)	47 (11.1)	21 (4.9)	127 (38.4)	9 (2.7)	195 (58.9)	105 (27.2)	9 (2.3)	272 (70.5)	57 (16.4)	7 (2.0)	283 (81.6)	50–75	<0.01
Livestock meat and poultry	242 (56.9)	127 (29.9)	56 (13.2)	159 (48.0)	108 (32.6)	64 (19.3)	140 (36.3)	127 (32.9)	119 (30.8)	64 (18.4)	67 (19.3)	216 (62.2)	50–75	<0.01
Eggs	302 (71.1)	80 (18.8)	43 (10.1)	219 (66.2)	71 (21.5)	41 (12.4)	226 (58.5)	110 (28.5)	50 (13.0)	170 (49.0)	82 (23.6)	95 (27.4)	50	<0.01
Milk and its products	367 (86.4)	43 (10.1)	15 (3.5)	237 (71.6)	78 (23.6)	16 (4.8)	221 (57.3)	126 (32.6)	39 (10.1)	133 (38.3)	165 (47.6)	49 (14.1)	300–500	<0.01
Soybean and its products	340 (80.0)	19 (4.5)	66 (15.1)	243 (73.4)	15 (4.5)	73 (22.1)	264 (68.4)	9 (2.3)	113 (29.3)	208 (59.9)	27 (7.8)	112 (32.3)	20	<0.01
Nuts	263 (61.9)	3 (0.7)	159 (37.4)	127 (38.4)	9 (2.7)	195 (58.9)	105 (27.2)	9 (2.3)	272 (70.5)	57 (16.4)	7 (2.0)	283 (81.6)	10	<0.01

^1^ Values are presented as number and percentage (*n* (%)). CDGCI–PW, Chinese dietary guidelines compliance index for pregnant women. ^2^ Below rec: The number and percentage of study participants whose food intakes are below the recommendation. ^3^ Within rec: The number and percentage of study participants whose food intakes are within the recommendation. ^4^ Above rec: The number and percentage of study participants whose food intakes are above the recommendation. ^5^ Rec: recommendation from Chinese balanced dietary pagoda for pregnant women.

**Table 5:** Associations between CDGCI-PW scores and risks of pregnancy complications ^1^ (N = 1489).

Characteristics	Value *n* (%)	Unadjusted Model, OR (95% CI)	Multivariable Model ^2^, OR (95% CI)
Gestational hypertension	71 (4.8)	0.26 (0.19,0.35) *	0.30 (0.20,0.37) *
Gestational diabetes mellitus	163 (10.9)	0.37 (0.30,0.45) *	0.38 (0.31,0.48) *

^1^ Values presented as number and percentage (*n* (%)) or logistic regression coefficients (95% CIs) for categorical variables per 10-point increment in CDGCI–PW scores. * *p* < 0.01. CDGCI–PW, Chinese dietary guidelines compliance index for pregnant women. ^2^ The multivariable model was adjusted for maternal age, pre-pregnancy BMI, weight gain in the second trimester, educational level, and household income.

## Data Availability

The data presented in this study are available on request from the corresponding author.

## Supplementary Materials

The following are available online at https://www.mdpi.com/2072-6643/13/3/829/s1, Table S1: Key recommendations of dietary guidelines for pregnant women and components and scoring criterion of CDGCI-PW1 (the first trimester); Table S2: Key recommendations of dietary guidelines for pregnant women and components and scoring criterion of CDGCI-PW1 (the third trimesters); Table S3: Classification of food items (under each food categories); Figure S1: Chinese Balanced Dietary Pagoda for Pregnant women in the second trimester.
