# Supplementary material for: Association between Chinese Dietary Guidelines Compliance Index for Pregnant Women and Risks of Pregnancy Complications in the Tongji Maternal and Child Health Cohort
Source: Nutrients. 2021 Mar 3;13(3):829. doi: 10.3390/nu13030829 (PMC7999266; doi:10.3390/nu13030829)
Supplement: Supplementary file 1 [file nutrients-13-00829-s001.pdf]

Table S1 Key recommendations of dietary guidelines for pregnant women and components and scoring criterion of CDGCI-PW[1] (the first trimester)

Key recommendation	Components of CDGCI-PW	Scoring criterion	Range of score
Eat a variety of foods, mainly cereals and their products;<br>A balanced diet includes 11 categories of foods per week and 12 kinds of foods per day;<br>Eat a balanced diet with no less than 130 g of carbohydrates per day;	1. How many categories of food do you eat per week on average?	A. Less than or equal to 6 categories	0 points
		B. Range from 7 to 10 categories	5 to 20 points (5 points for each additional category)
		C. More than or equal to 11 categories	25 points
	2. How many kinds of foods do you eat per day on average?	A. Less than or equal to 4 kinds	0 points
		B. Range from 5 to 11 kinds	1 to 7 points (1 points for each additional kind)
		C. Range from 11 to 20 kinds	9 to 25 points (2 points for each additional kind)
		D. More than or equal to 20 kinds	25 points
	3. Does your daily intake of staple food reach 150g on average?	A. No	0 points
		B. Yes	5 points
Folic acid should be taken orally at 400 g/d and green-leafy and colored (red and yellow) vegetables should be taken.	4. Do you take 400μg of folic acid per day on average?	A. Yes	0 points
		B. No	5 points
	5. Do you eat more than 200g of green-leafy and colored vegetables (red and yellow) per day on average? (raw weight)	A. No	0 points
		B. Yes	5 points
Ensure adequate intake of milk and its products, and soybeans and its products.	6. How often do you drink milk and its products per week on average? Dairy intake is considered to be significant when servings are equivalent to 250ml of fresh liquid milk each time.	A. Less than or equal to once a week	0 points
		B. Range from 2 to 4 times per week	1 to 3 points (1 point for each additional time per week)
		C. More than or equal to 5 times per week	4 points
	7. How often do you eat soybeans and soybean products per week on average? The intake of soybeans	A. Less than or equal to once a week	0 points
		B. Range from 2 to 4 times per week	1 to 3 points (1 point for each

	products is considered to be significant when servings are the equivalent of up to 15g of dry soybeans each time.		additional time per week)
		C. More than or equal to 5 times per week	4 points
Eat appropriate amounts of lean meat (livestock and poultry meat), aquatic products (fish, shrimp, and shellfish), eggs, and nuts.	8. Do you eat 100g of lean meat (livestock and poultry meat), aquatic products (fish, shrimp, and shellfish), or eggs per day on average?	A. No	0 points
		B. Yes	4 points
	9. How often do you eat nuts per week on average? Nuts intake is considered to be significant when servings are the equivalent of up to 10 g of dry nuts each time.	A. Less than or equal to once a week	0 points
		B. Range from 2 to 4 times per week	1 to 3 points (1 point for each additional time per week)
		C. More than or equal to 5 times per week	4 points
Eat iron-rich foods such as animal blood or liver once or twice a week	10. How often do you eat animal blood and liver per week on average? The intake of animal blood and liver is considered to be significant when it is reach to 20 to 50g each time？	A. Never eat	0 points
		B. 1 time per week	3 points
		C. More than or equal to 2 times per week	4 points
Eat iodized table salt and iodine rich seafood such as deep sea-dwelling fish twice or three a week	11. Do you eat iodized table salt every day？	A. No	0 points
		B. Yes	5 points
	12. How often do you eat iodine rich seafood per week on average? This includes kelp, nori, undaria pinnatifida, shellfish, sea fish, etc.	A. Never eat	0 points
		B. Range from 1 to 4 times per week	1 to 4 points (1 point for each additional time per week)
		C. More than or equal to 5 times per week	4 points
Develop healthy eating habits	13. Do you often eat foods high in oil, salt and sugar？	A. Yes	0 points
		B. No	6 points

[1]CDGCI–PW, Chinese dietary guidelines compliance index for pregnant women.

Table S2 Key recommendations of dietary guidelines for pregnant women and components and scoring criterion of CDGCI-PW[1] (the third trimesters)

Key recommendation	Components of CDGCI-PW	Scoring criterion	Range of score
Eat a variety of foods, mainly cereals and their products (A balanced diet includes 11 categories of foods per week and 12 kinds of foods per day. Whole grains and beans other than soybeans should form no less than one-third of the total intake of staple food)	1. How many categories of food do you eat per week on average?	A. Less than or equal to 6 categories	0 points
		B. Range from7 to 10 categories	5 to 20 points (5 points for each additional category)
		C. More than or equal to 11 categories	25 points
	2. How many kinds of foods do you eat per day on average?	A. Less than or equal to 4 kinds	0 points
		B. Range from 5 to 11 kinds	1 to 7 points (1 points for each additional kind)
		C. Range from 11 to 20 kinds	7 to 25 points (2 points for each additional kind)
		D. More than or equal to 20 kinds	25 points
	3. Does your daily intake of staple food reach 200g on average?	A. No	0 points
		B. Yes	4 points
	4. Can whole grains and beans other than soybeans account for more than one-third of your staple food intake?	A. No	0 points
		B. Yes	4 points
Ensure adequate intake of vegetables, milk and its products, soybeans and its products, and nuts. Among them, the intake of green-leafy and colored vegetables (red and yellow) should reach 200 g per day.	5. Do you eat more than 200g of green-leafy and colored vegetables (red and yellow) per day on average? (raw weight)	A. No	0 points
		B. Yes	5 points
	6. How often do you drink milk and its products per week on average? Dairy intake is considered to be significant when servings are equivalent to 250ml of fresh liquid milk each time.	A. Less than or equal to once a week	0 points
		B. Range from 2 to 4 times per week	1 to 3 points (1 point for each additional time per week)
		C. More than or equal to 5 times per week	4 points
	7. How often do you eat soybeans and soybean products per week on average? The intake of soybeans	A. Less than or equal to once a week	0 points
		B. Range from 2 to 4 times per week	1 to 3 points (1 point for each

	products is considered to be significant when servings are the equivalent of up to 15 g of dry soybeans each time.		additional time per week)
		C. More than or equal to 5 times per week	4 points
Eat appropriate amounts of lean meat (livestock and poultry meat), aquatic products (fish, shrimp, and shellfish), eggs, and nuts. Eat iron-rich foods such as animal blood or liver once or twice a week.	8. Do you eat 125g of lean meat (livestock and poultry meat), aquatic products (fish, shrimp, and shellfish), or eggs per day on average?	A. No	0 points
		B. Yes	5 points
	9. How often do you eat nuts per week on average? Nuts intake is considered to be significant when servings are the equivalent of up to 10g of dry nuts each time.	A. Less than or equal to once a week	0 points
		B. Range from 2 to 4 times per week	1 to 3 points (1 point for each additional time per week)
		C. More than or equal to 5 times per week	4 points
	10. How often do you eat animal blood and liver per week on average? The intake of animal blood and liver is considered to be significant when it is reach to 20 to 50g each time.	A. Never eat	0 points
		B. 1 time per week	3 points
		C. More than or equal to 2 times per week	5 points
Eat iodized table salt and iodine rich seafood.	11. Do you eat iodized table salt every day?	A. No	0 points
		B. Yes	4 points
	12. How often do you eat iodine rich seafood per week on average? This includes kelp, nori, undaria pinnatifida, shellfish, sea fish, etc.	A. Never eat	0 points
		B. Range from 1 to 4 times per week	1 to 4 points (1 point for each additional time per week)
		C. More than or equal to 5 times per week	5 points
Develop healthy eating habits	13. Do you often eat foods high in oil, salt and sugar?	A. Yes	0 points
		B. No	6 points

[1]CDGCI–PW, Chinese dietary guidelines compliance index for pregnant women.

Table S3: Classification of food items (under each food categories)

Food categories (Number of food items)	Food name*
Cereals and their products, potatoes and beans other than soybeans (6)	Rice and its products, Wheat flour and its products, Coarse Cereals, Potatoes and its products, Snacks, Beans other than soybeans
Vegetables (12)	Dark-colored leafy/flower vegetables, Light-colored leafy / flower vegetables, Root and stem vegetables, Radish, Allium vegetables, Leguminous vegetables and sprout, Cucurbitaceous and solanaceous vegetables, Tomatoes, Pepper, Mushrooms, Fungi, Algae
Fruits (10)	Citrus, Pome fruits, Banana, Berry, Melons, Drupe (one-seeded) fruits, Subtropical fruits, Sugarcane, Dried/preserved fruits, Jam
Aquatic products (4)	Freshwater fish, Marine fish, Shellfish, Mollusks
Livestock meat and poultry (9)	Pork / Pork ribs, Beef, Lamb, Poultry meat, Liver / Blood, Other viscera
Eggs (1)	Eggs and their products
Milk and its Products (3)	Milk, Yogurt, Formula milk powder
Soybean and its products (1)	Soybean and its products
Nuts (10)	Nuts rich in oil, Nuts rich in starch
Cooking oil (2)	Vegetable oil, Animal oil
Salt (2)	Iodized salt, Non iodized salt

* The food name comes from China food composition tables (6th edition)

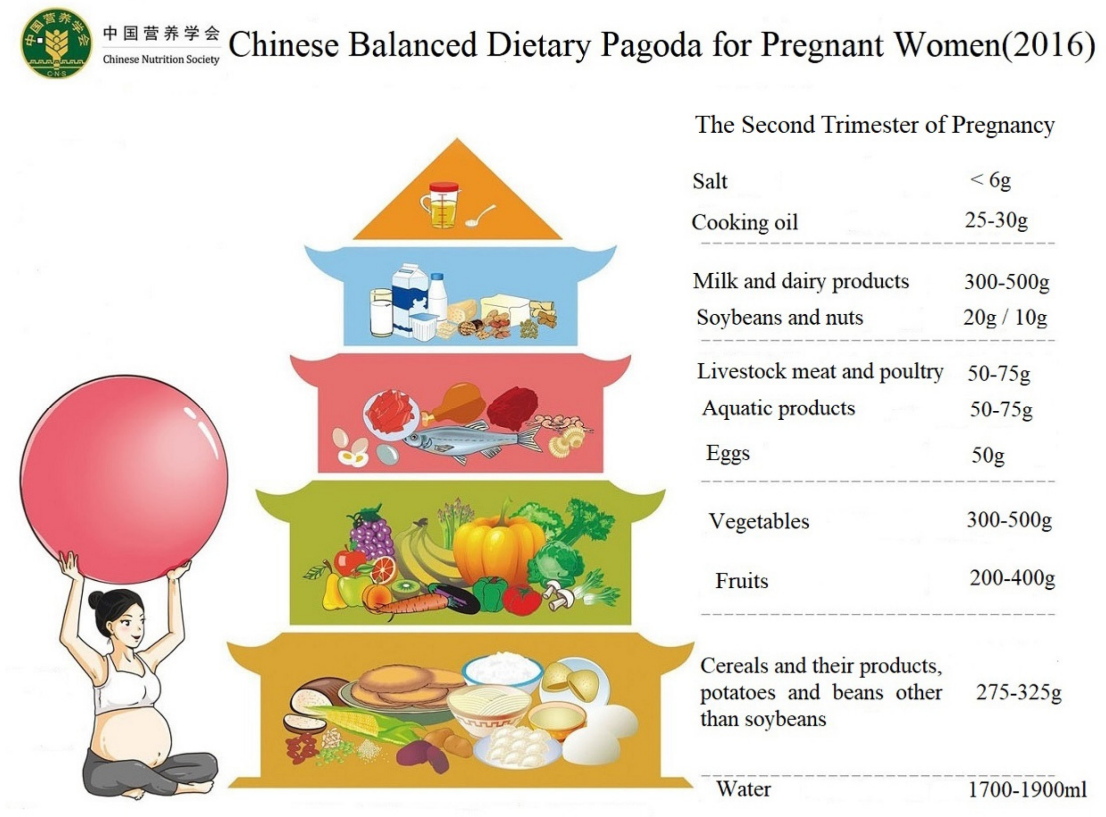

Figure S1 Chinese Balanced Dietary Pagoda for Pregnant women in the second trimester
